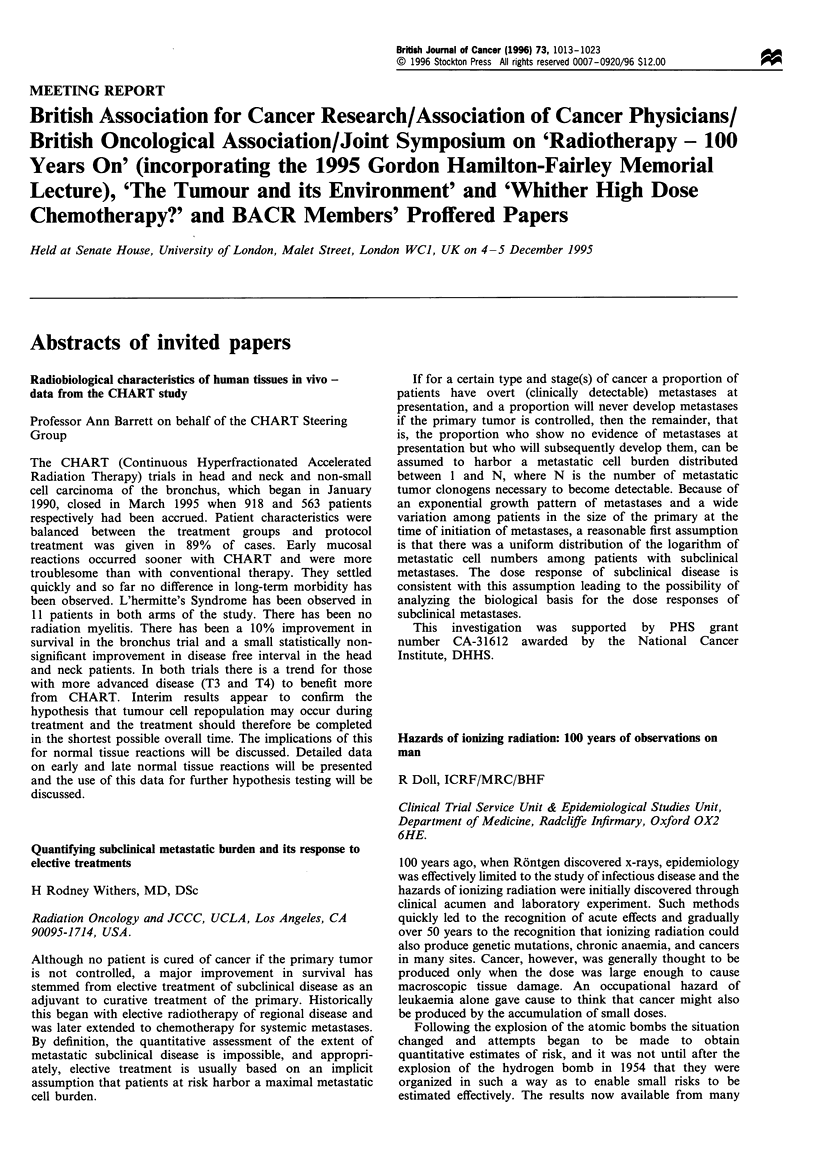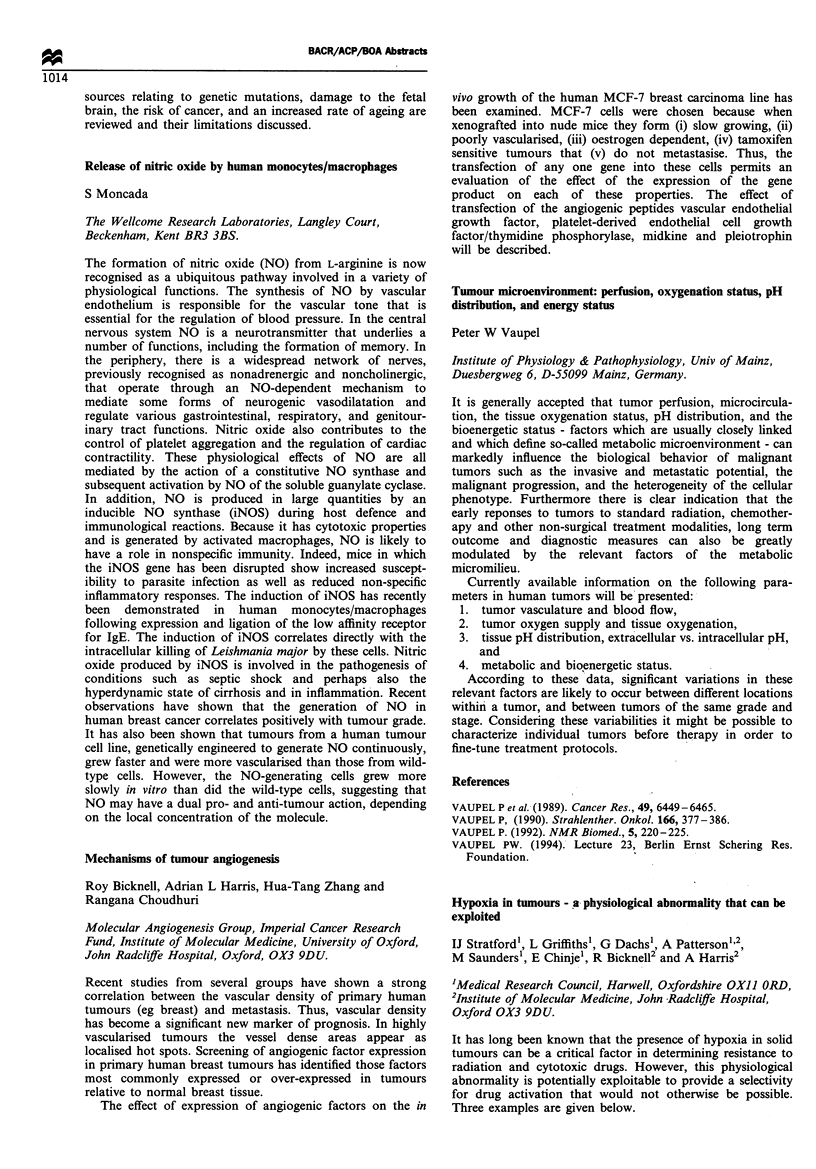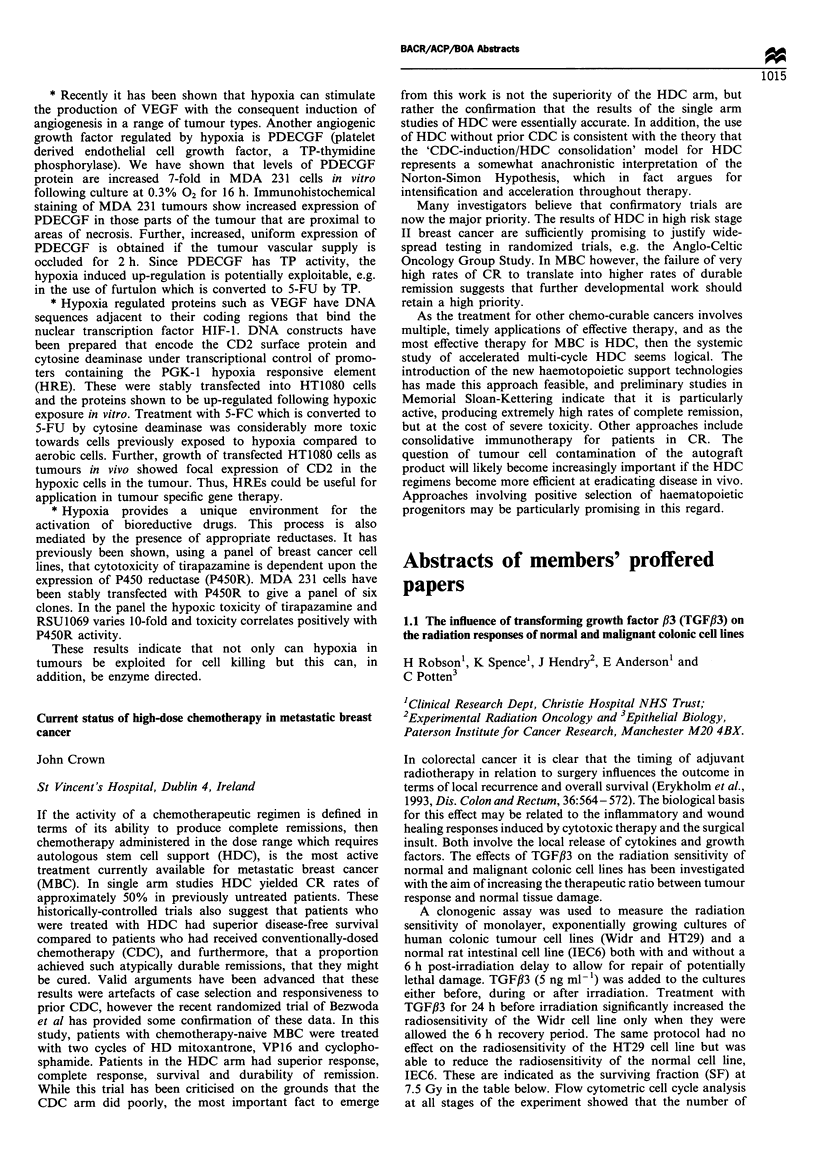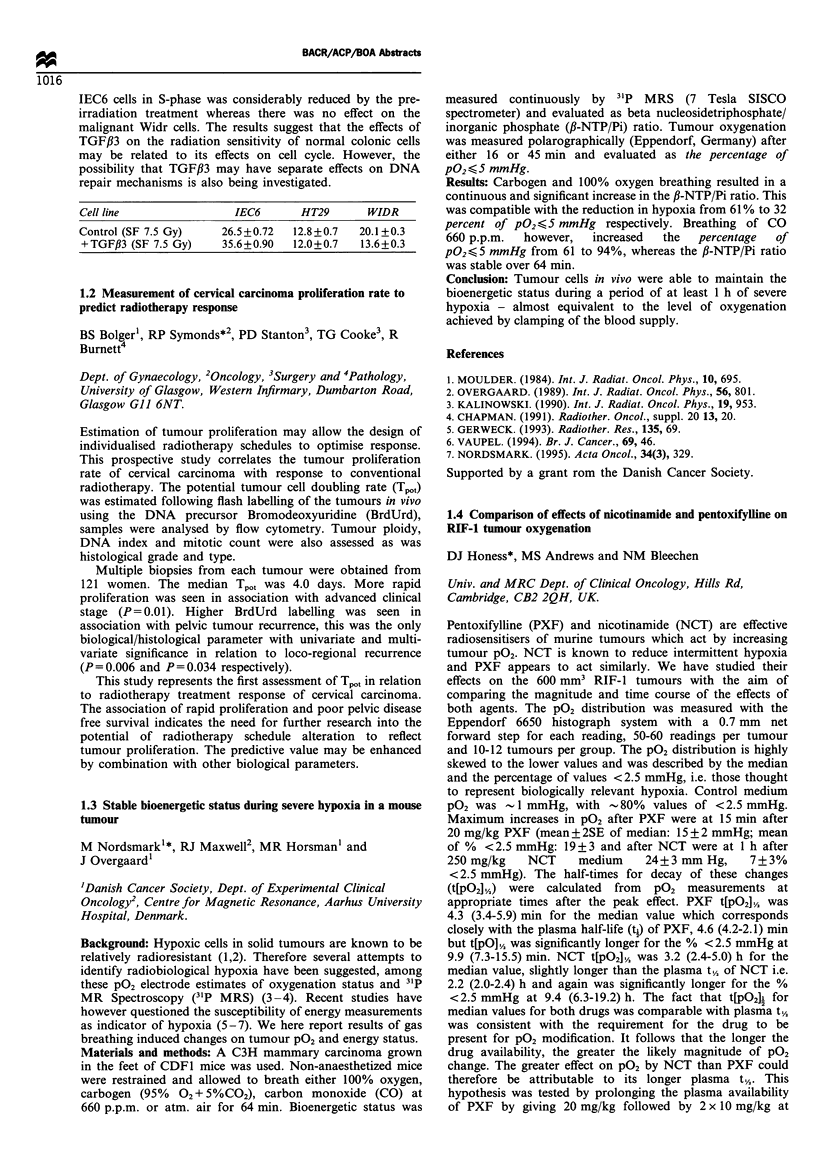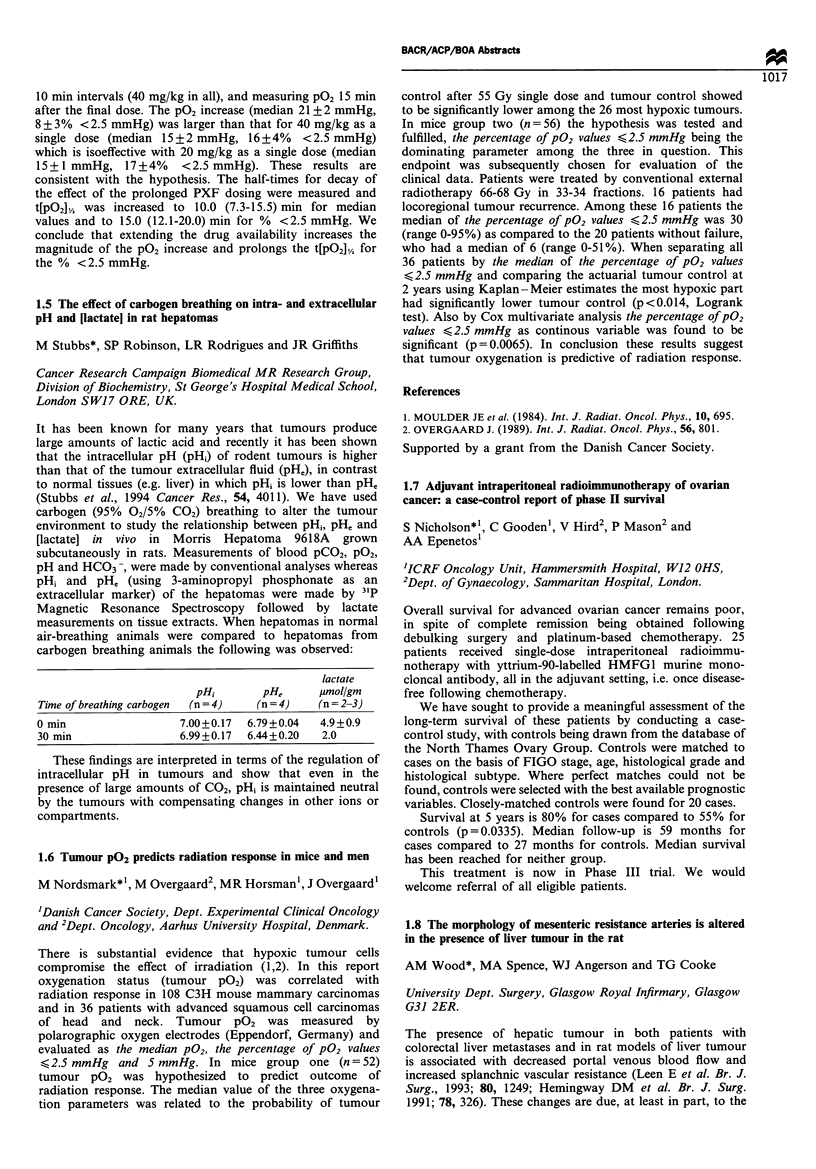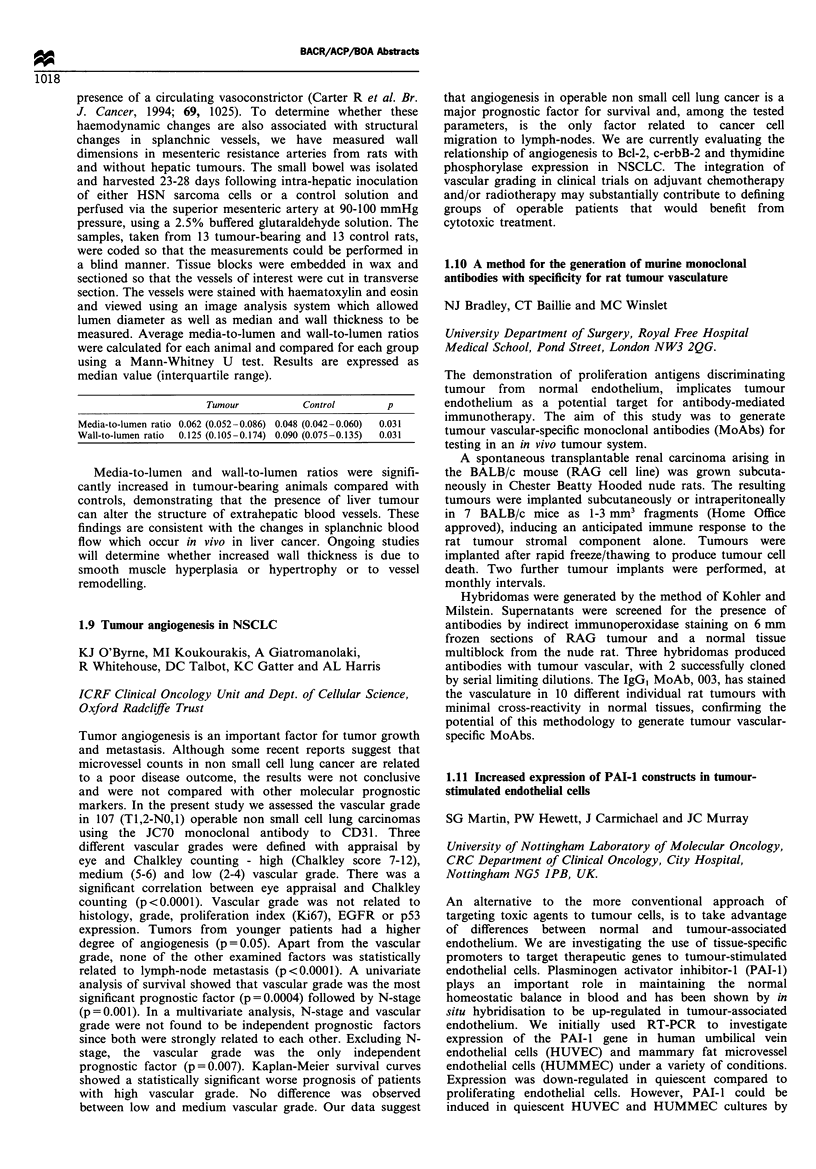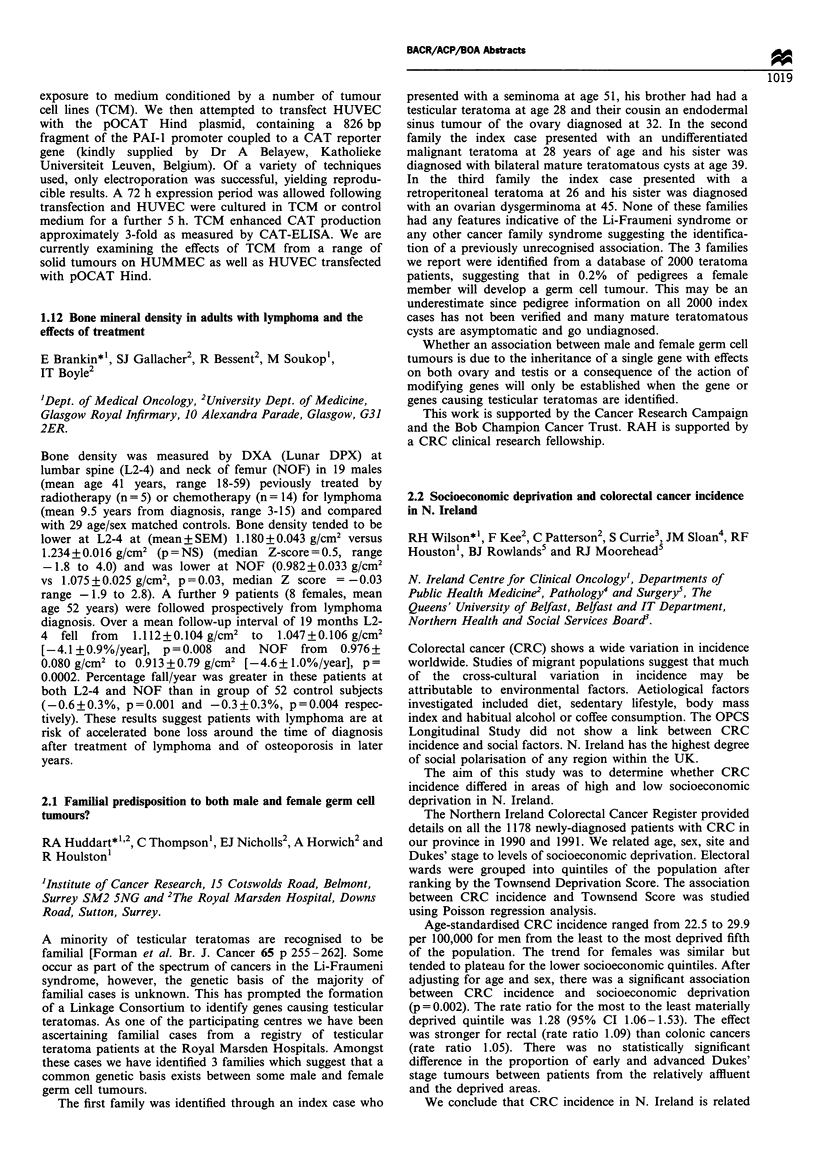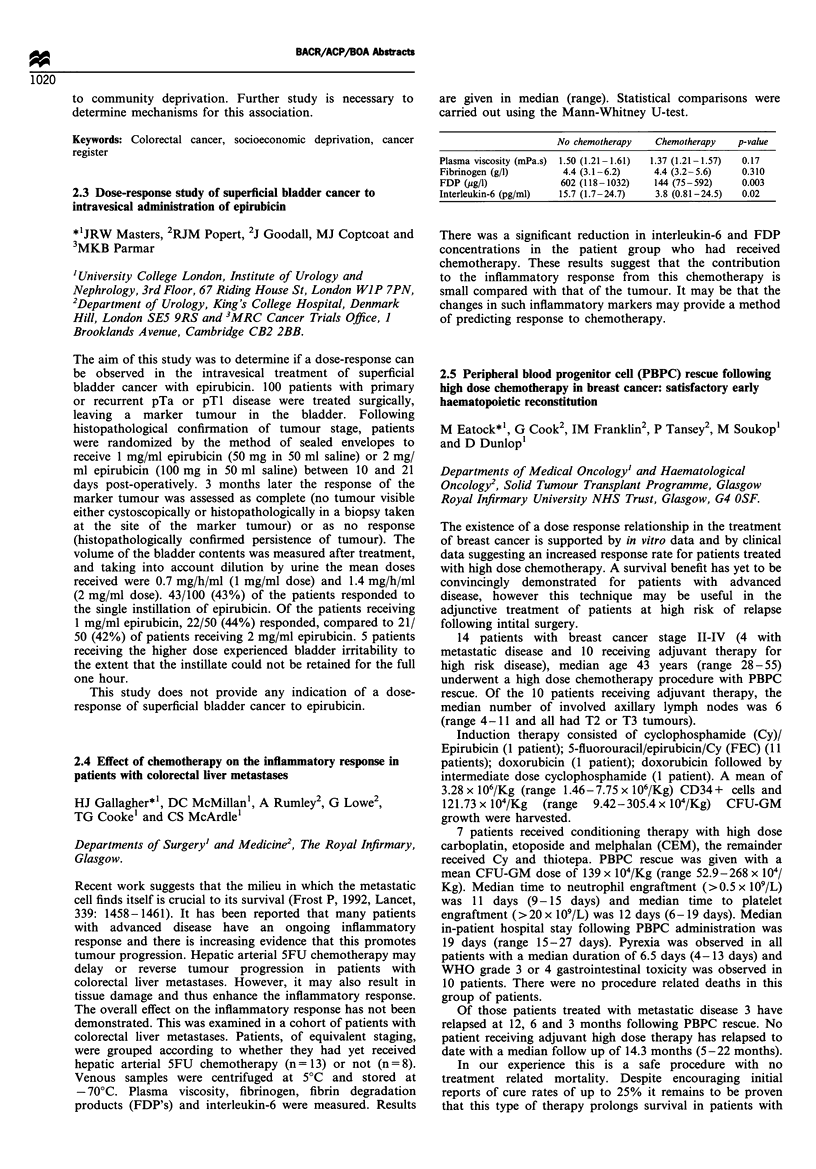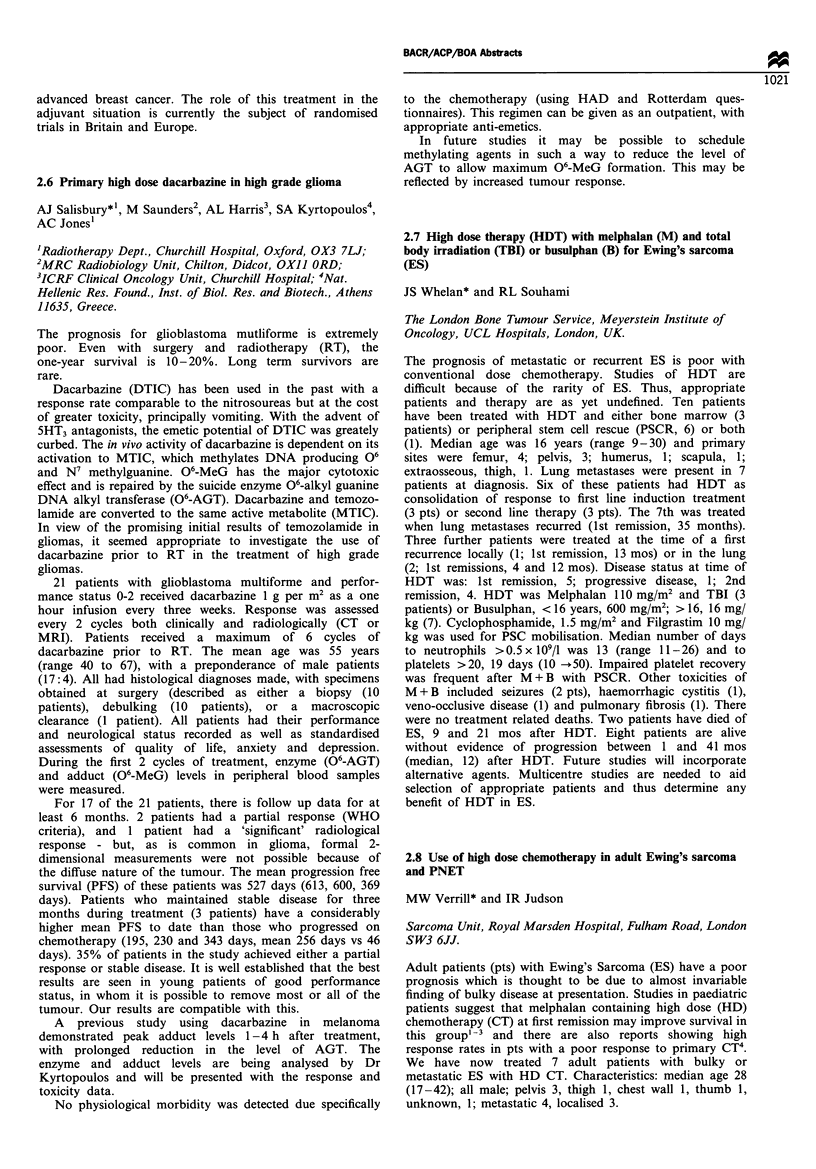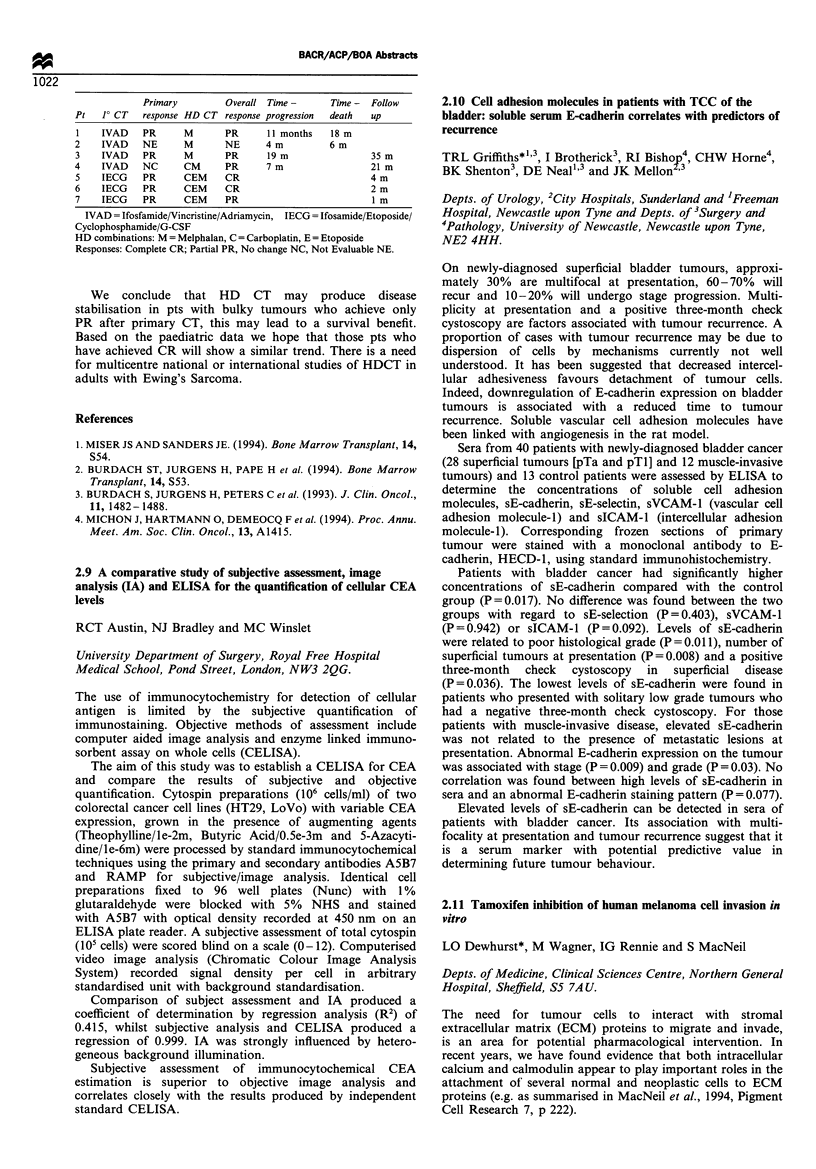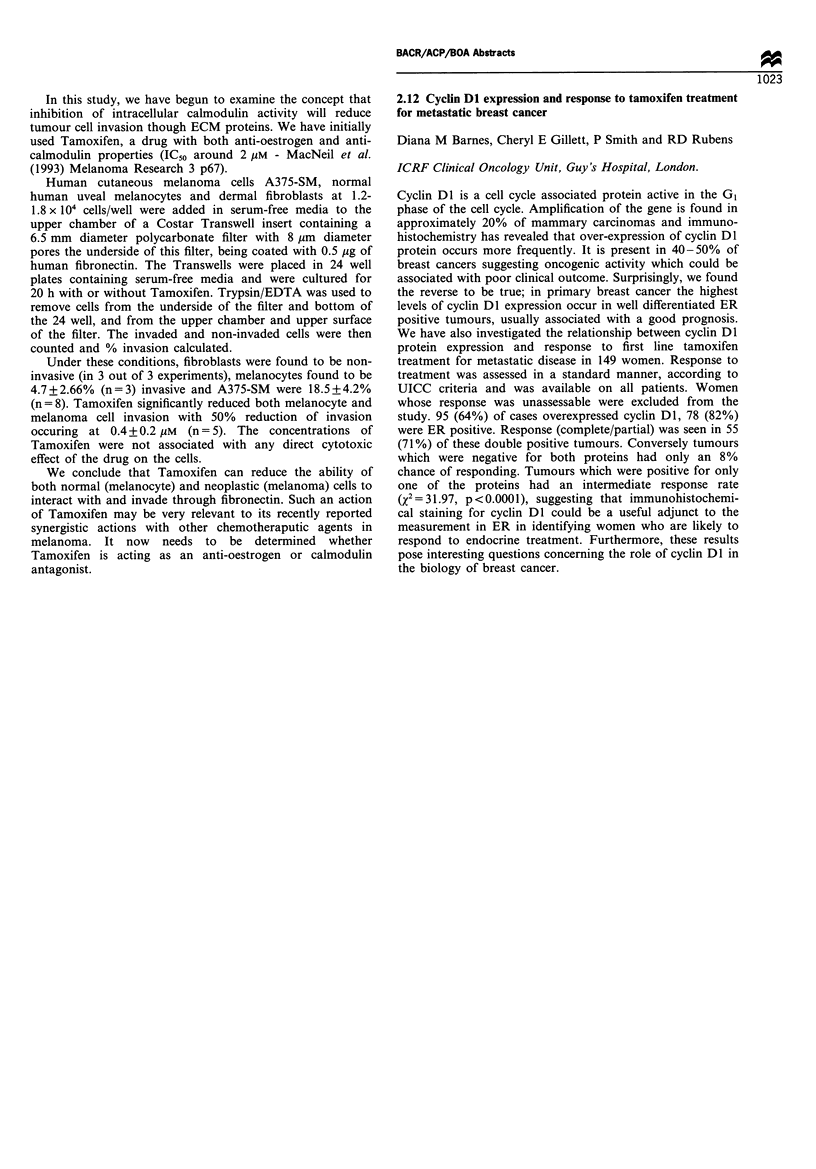# British Association for Cancer Research/Association of Cancer Physicians/British Oncological Association/Joint Symposium on `Radiotherapy - 100 Years On' (incorporating the 1995 Gordon Hamilton-Fairley Memorial Lecture), 'The Tumour and its Environment' and 'Whither High Dose Chemotherapy?' and BACR Members' Proffered Papers

**Published:** 1996-04

**Authors:** 


British Journal of Cancer (1996) 73, 1013-1023
© 1996 Stockton Press All rights reserved 0007-0920/96 $12.00

MEETING REPORT

### British Association for Cancer Research/Association of Cancer Physicians/ British Oncological Association/Joint Symposium on 'Radiotherapy – 100 Years On' (incorporating the 1995 Gordon Hamilton-Fairley Memorial Lecture), 'The Tumour and its Environment' and 'Whither High Dose Chemotherapy?' and BACR Members' Proffered Papers

*Held at Senate House, University of London, Malet Street, London WC1, UK on 4–5 December 1995*

### Abstracts of invited papers

### Radiobiological characteristics of human tissues in vivo – data from the CHART study

Professor Ann Barrett on behalf of the CHART Steering Group

The CHART (Continuous Hyperfractionated Accelerated Radiation Therapy) trials in head and neck and non-small cell carcinoma of the bronchus, which began in January 1990, closed in March 1995 when 918 and 563 patients respectively had been accrued. Patient characteristics were balanced between the treatment groups and protocol treatment was given in 89% of cases. Early mucosal reactions occurred sooner with CHART and were more troublesome than with conventional therapy. They settled quickly and so far no difference in long-term morbidity has been observed. L'hermitte's Syndrome has been observed in 11 patients in both arms of the study. There has been no radiation myelitis. There has been a 10% improvement in survival in the bronchus trial and a small statistically non-significant improvement in disease free interval in the head and neck patients. In both trials there is a trend for those with more advanced disease (T3 and T4) to benefit more from CHART. Interim results appear to confirm the hypothesis that tumour cell repopulation may occur during treatment and the treatment should therefore be completed in the shortest possible overall time. The implications of this for normal tissue reactions will be discussed. Detailed data on early and late normal tissue reactions will be presented and the use of this data for further hypothesis testing will be discussed.

### Quantifying subclinical metastatic burden and its response to elective treatments

H Rodney Withers, MD, DSc

*Radiation Oncology and JCCC, UCLA, Los Angeles, CA 90095-1714, USA.*

Although no patient is cured of cancer if the primary tumor is not controlled, a major improvement in survival has stemmed from elective treatment of subclinical disease as an adjuvant to curative treatment of the primary. Historically this began with elective radiotherapy of regional disease and was later extended to chemotherapy for systemic metastases. By definition, the quantitative assessment of the extent of metastatic subclinical disease is impossible, and appropriately, elective treatment is usually based on an implicit assumption that patients at risk harbor a maximal metastatic cell burden.

If for a certain type and stage(s) of cancer a proportion of patients have overt (clinically detectable) metastases at presentation, and a proportion will never develop metastases if the primary tumor is controlled, then the remainder, that is, the proportion who show no evidence of metastases at presentation but who will subsequently develop them, can be assumed to harbor a metastatic cell burden distributed between 1 and N, where N is the number of metastatic tumor clonogens necessary to become detectable. Because of an exponential growth pattern of metastases and a wide variation among patients in the size of the primary at the time of initiation of metastases, a reasonable first assumption is that there was a uniform distribution of the logarithm of metastatic cell numbers among patients with subclinical metastases. The dose response of subclinical disease is consistent with this assumption leading to the possibility of analyzing the biological basis for the dose responses of subclinical metastases.

This investigation was supported by PHS grant number CA-31612 awarded by the National Cancer Institute, DHHS.

### Hazards of ionizing radiation: 100 years of observations on man

R Doll, ICRF/MRC/BHF

*Clinical Trial Service Unit & Epidemiological Studies Unit, Department of Medicine, Radcliffe Infirmary, Oxford OX2 6HE.*

100 years ago, when Röntgen discovered x-rays, epidemiology was effectively limited to the study of infectious disease and the hazards of ionizing radiation were initially discovered through clinical acumen and laboratory experiment. Such methods quickly led to the recognition of acute effects and gradually over 50 years to the recognition that ionizing radiation could also produce genetic mutations, chronic anaemia, and cancers in many sites. Cancer, however, was generally thought to be produced only when the dose was large enough to cause macroscopic tissue damage. An occupational hazard of leukaemia alone gave cause to think that cancer might also be produced by the accumulation of small doses.

Following the explosion of the atomic bombs the situation changed and attempts began to be made to obtain quantitative estimates of risk, and it was not until after the explosion of the hydrogen bomb in 1954 that they were organized in such a way as to enable small risks to be estimated effectively. The results now available from many sources relating to genetic mutations, damage to the fetal brain, the risk of cancer, and an increased rate of ageing are reviewed and their limitations discussed.

### Release of nitric oxide by human monocytes/macrophages

S Moncada

*The Wellcome Research Laboratories, Langley Court, Beckenham, Kent BR3 3BS.*

The formation of nitric oxide (NO) from L-arginine is now recognised as a ubiquitous pathway involved in a variety of physiological functions. The synthesis of NO by vascular endothelium is responsible for the vascular tone that is essential for the regulation of blood pressure. In the central nervous system NO is a neurotransmitter that underlies a number of functions, including the formation of memory. In the periphery, there is a widespread network of nerves, previously recognised as nonadrenergic and noncholinergic, that operate through an NO-dependent mechanism to mediate some forms of neurogenic vasodilatation and regulate various gastrointestinal, respiratory, and genitourinary tract functions. Nitric oxide also contributes to the control of platelet aggregation and the regulation of cardiac contractility. These physiological effects of NO are all mediated by the action of a constitutive NO synthase and subsequent activation by NO of the soluble guanylate cyclase. In addition, NO is produced in large quantities by an inducible NO synthase (iNOS) during host defence and immunological reactions. Because it has cytotoxic properties and is generated by activated macrophages, NO is likely to have a role in nonspecific immunity. Indeed, mice in which the iNOS gene has been disrupted show increased susceptibility to parasite infection as well as reduced non-specific inflammatory responses. The induction of iNOS has recently been demonstrated in human monocytes/macrophages following expression and ligation of the low affinity receptor for IgE. The induction of iNOS correlates directly with the intracellular killing of *Leishmania major* by these cells. Nitric oxide produced by iNOS is involved in the pathogenesis of conditions such as septic shock and perhaps also the hyperdynamic state of cirrhosis and in inflammation. Recent observations have shown that the generation of NO in human breast cancer correlates positively with tumour grade. It has also been shown that tumours from a human tumour cell line, genetically engineered to generate NO continuously, grew faster and were more vascularised than those from wild-type cells. However, the NO-generating cells grew more slowly *in vitro* than did the wild-type cells, suggesting that NO may have a dual pro- and anti-tumour action, depending on the local concentration of the molecule.

### Mechanisms of tumour angiogenesis

Roy Bicknell, Adrian L Harris, Hua-Tang Zhang and Rangana Choudhuri

*Molecular Angiogenesis Group, Imperial Cancer Research Fund, Institute of Molecular Medicine, University of Oxford, John Radcliffe Hospital, Oxford, OX3 9DU.*

Recent studies from several groups have shown a strong correlation between the vascular density of primary human tumours (eg breast) and metastasis. Thus, vascular density has become a significant new marker of prognosis. In highly vascularised tumours the vessel dense areas appear as localised hot spots. Screening of angiogenic factor expression in primary human breast tumours has identified those factors most commonly expressed or over-expressed in tumours relative to normal breast tissue.

The effect of expression of angiogenic factors on the *in vivo* growth of the human MCF-7 breast carcinoma line has been examined. MCF-7 cells were chosen because when xenografted into nude mice they form (i) slow growing, (ii) poorly vascularised, (iii) oestrogen dependent, (iv) tamoxifen sensitive tumours that (v) do not metastasise. Thus, the transfection of any one gene into these cells permits an evaluation of the effect of the expression of the gene product on each of these properties. The effect of transfection of the angiogenic peptides vascular endothelial growth factor, platelet-derived endothelial cell growth factor/thymidine phosphorylase, midkine and pleiotrophin will be described.

### Tumour microenvironment: perfusion, oxygenation status, pH distribution, and energy status

Peter W Vaupel

*Institute of Physiology & Pathophysiology, Univ of Mainz, Duesbergweg 6, D-55099 Mainz, Germany.*

It is generally accepted that tumor perfusion, microcirculation, the tissue oxygenation status, pH distribution, and the bioenergetic status - factors which are usually closely linked and which define so-called metabolic microenvironment - can markedly influence the biological behavior of malignant tumors such as the invasive and metastatic potential, the malignant progression, and the heterogeneity of the cellular phenotype. Furthermore there is clear indication that the early reponses to tumors to standard radiation, chemotherapy and other non-surgical treatment modalities, long term outcome and diagnostic measures can also be greatly modulated by the relevant factors of the metabolic micromilieu.

Currently available information on the following parameters in human tumors will be presented:

1. tumor vasculature and blood flow,
2. tumor oxygen supply and tissue oxygenation,
3. tissue pH distribution, extracellular vs. intracellular pH, and
4. metabolic and bioenergetic status.

According to these data, significant variations in these relevant factors are likely to occur between different locations within a tumor, and between tumors of the same grade and stage. Considering these variabilities it might be possible to characterize individual tumors before therapy in order to fine-tune treatment protocols.

#### References

VAUPEL P *et al.* (1989). *Cancer Res.*, **49**, 6449–6465.
VAUPEL P, (1990). *Strahlenther. Onkol.* **166**, 377–386.
VAUPEL P. (1992). *NMR Biomed.*, **5**, 220–225.
VAUPEL PW. (1994). Lecture 23, Berlin Ernst Schering Res. Foundation.

### Hypoxia in tumours - a physiological abnormality that can be exploited

IJ Stratford[1], L Griffiths[1], G Dachs[1], A Patterson[1,2], M Saunders[1], E Chinje[1], R Bicknell[2] and A Harris[2]

*[1]Medical Research Council, Harwell, Oxfordshire OX11 0RD, [2]Institute of Molecular Medicine, John Radcliffe Hospital, Oxford OX3 9DU.*

It has long been known that the presence of hypoxia in solid tumours can be a critical factor in determining resistance to radiation and cytotoxic drugs. However, this physiological abnormality is potentially exploitable to provide a selectivity for drug activation that would not otherwise be possible. Three examples are given below.

* Recently it has been shown that hypoxia can stimulate the production of VEGF with the consequent induction of angiogenesis in a range of tumour types. Another angiogenic growth factor regulated by hypoxia is PDECGF (platelet derived endothelial cell growth factor, a TP-thymidine phosphorylase). We have shown that levels of PDECGF protein are increased 7-fold in MDA 231 cells *in vitro* following culture at 0.3% $O_2$ for 16 h. Immunohistochemical staining of MDA 231 tumours show increased expression of PDECGF in those parts of the tumour that are proximal to areas of necrosis. Further, increased, uniform expression of PDECGF is obtained if the tumour vascular supply is occluded for 2 h. Since PDECGF has TP activity, the hypoxia induced up-regulation is potentially exploitable, e.g. in the use of furtulon which is converted to 5-FU by TP.

* Hypoxia regulated proteins such as VEGF have DNA sequences adjacent to their coding regions that bind the nuclear transcription factor HIF-1. DNA constructs have been prepared that encode the CD2 surface protein and cytosine deaminase under transcriptional control of promoters containing the PGK-1 hypoxia responsive element (HRE). These were stably transfected into HT1080 cells and the proteins shown to be up-regulated following hypoxic exposure *in vitro*. Treatment with 5-FC which is converted to 5-FU by cytosine deaminase was considerably more toxic towards cells previously exposed to hypoxia compared to aerobic cells. Further, growth of transfected HT1080 cells as tumours *in vivo* showed focal expression of CD2 in the hypoxic cells in the tumour. Thus, HREs could be useful for application in tumour specific gene therapy.

* Hypoxia provides a unique environment for the activation of bioreductive drugs. This process is also mediated by the presence of appropriate reductases. It has previously been shown, using a panel of breast cancer cell lines, that cytotoxicity of tirapazamine is dependent upon the expression of P450 reductase (P450R). MDA 231 cells have been stably transfected with P450R to give a panel of six clones. In the panel the hypoxic toxicity of tirapazamine and RSU1069 varies 10-fold and toxicity correlates positively with P450R activity.

These results indicate that not only can hypoxia in tumours be exploited for cell killing but this can, in addition, be enzyme directed.

**Current status of high-dose chemotherapy in metastatic breast cancer**

John Crown

*St Vincent's Hospital, Dublin 4, Ireland*

If the activity of a chemotherapeutic regimen is defined in terms of its ability to produce complete remissions, then chemotherapy administered in the dose range which requires autologous stem cell support (HDC), is the most active treatment currently available for metastatic breast cancer (MBC). In single arm studies HDC yielded CR rates of approximately 50% in previously untreated patients. These historically-controlled trials also suggest that patients who were treated with HDC had superior disease-free survival compared to patients who had received conventionally-dosed chemotherapy (CDC), and furthermore, that a proportion achieved such atypically durable remissions, that they might be cured. Valid arguments have been advanced that these results were artefacts of case selection and responsiveness to prior CDC, however the recent randomized trial of Bezwoda *et al* has provided some confirmation of these data. In this study, patients with chemotherapy-naive MBC were treated with two cycles of HD mitoxantrone, VP16 and cyclophosphamide. Patients in the HDC arm had superior response, complete response, survival and durability of remission. While this trial has been criticised on the grounds that the CDC arm did poorly, the most important fact to emerge from this work is not the superiority of the HDC arm, but rather the confirmation that the results of the single arm studies of HDC were essentially accurate. In addition, the use of HDC without prior CDC is consistent with the theory that the 'CDC-induction/HDC consolidation' model for HDC represents a somewhat anachronistic interpretation of the Norton-Simon Hypothesis, which in fact argues for intensification and acceleration throughout therapy.

Many investigators believe that confirmatory trials are now the major priority. The results of HDC in high risk stage II breast cancer are sufficiently promising to justify widespread testing in randomized trials, e.g. the Anglo-Celtic Oncology Group Study. In MBC however, the failure of very high rates of CR to translate into higher rates of durable remission suggests that further developmental work should retain a high priority.

As the treatment for other chemo-curable cancers involves multiple, timely applications of effective therapy, and as the most effective therapy for MBC is HDC, then the systemic study of accelerated multi-cycle HDC seems logical. The introduction of the new haemotopoietic support technologies has made this approach feasible, and preliminary studies in Memorial Sloan-Kettering indicate that it is particularly active, producing extremely high rates of complete remission, but at the cost of severe toxicity. Other approaches include consolidative immunotherapy for patients in CR. The question of tumour cell contamination of the autograft product will likely become increasingly important if the HDC regimens become more efficient at eradicating disease in vivo. Approaches involving positive selection of haematopoietic progenitors may be particularly promising in this regard.

### Abstracts of members' proffered papers

**1.1 The influence of transforming growth factor $\beta$3 (TGF$\beta$3) on the radiation responses of normal and malignant colonic cell lines**

H Robson[1], K Spence[1], J Hendry[2], E Anderson[1] and C Potten[3]

[1]*Clinical Research Dept, Christie Hospital NHS Trust;* [2]*Experimental Radiation Oncology and* [3]*Epithelial Biology, Paterson Institute for Cancer Research, Manchester M20 4BX.*

In colorectal cancer it is clear that the timing of adjuvant radiotherapy in relation to surgery influences the outcome in terms of local recurrence and overall survival (Erykholm *et al.*, 1993, *Dis. Colon and Rectum*, 36:564–572). The biological basis for this effect may be related to the inflammatory and wound healing responses induced by cytotoxic therapy and the surgical insult. Both involve the local release of cytokines and growth factors. The effects of TGF$\beta$3 on the radiation sensitivity of normal and malignant colonic cell lines has been investigated with the aim of increasing the therapeutic ratio between tumour response and normal tissue damage.

A clonogenic assay was used to measure the radiation sensitivity of monolayer, exponentially growing cultures of human colonic tumour cell lines (Widr and HT29) and a normal rat intestinal cell line (IEC6) both with and without a 6 h post-irradiation delay to allow for repair of potentially lethal damage. TGF$\beta$3 (5 ng $ml^{-1}$) was added to the cultures either before, during or after irradiation. Treatment with TGF$\beta$3 for 24 h before irradiation significantly increased the radiosensitivity of the Widr cell line only when they were allowed the 6 h recovery period. The same protocol had no effect on the radiosensitivity of the HT29 cell line but was able to reduce the radiosensitivity of the normal cell line, IEC6. These are indicated as the surviving fraction (SF) at 7.5 Gy in the table below. Flow cytometric cell cycle analysis at all stages of the experiment showed that the number of

IEC6 cells in S-phase was considerably reduced by the pre-irradiation treatment whereas there was no effect on the malignant Widr cells. The results suggest that the effects of TGF$\beta$3 on the radiation sensitivity of normal colonic cells may be related to its effects on cell cycle. However, the possibility that TGF$\beta$3 may have separate effects on DNA repair mechanisms is also being investigated.

| *Cell line* | *IEC6* | *HT29* | *WIDR* |
|---|---|---|---|
| Control (SF 7.5 Gy) | 26.5±0.72 | 12.8±0.7 | 20.1±0.3 |
| +TGF$\beta$3 (SF 7.5 Gy) | 35.6±0.90 | 12.0±0.7 | 13.6±0.3 |

### 1.2 Measurement of cervical carcinoma proliferation rate to predict radiotherapy response

BS Bolger[1], RP Symonds*[2], PD Stanton[3], TG Cooke[3], R Burnett[4]

*Dept. of Gynaecology, [2]Oncology, [3]Surgery and [4]Pathology, University of Glasgow, Western Infirmary, Dumbarton Road, Glasgow G11 6NT.*

Estimation of tumour proliferation may allow the design of individualised radiotherapy schedules to optimise response. This prospective study correlates the tumour proliferation rate of cervical carcinoma with response to conventional radiotherapy. The potential tumour cell doubling rate ($T_{pot}$) was estimated following flash labelling of the tumours *in vivo* using the DNA precursor Bromodeoxyuridine (BrdUrd), samples were analysed by flow cytometry. Tumour ploidy, DNA index and mitotic count were also assessed as was histological grade and type.

Multiple biopsies from each tumour were obtained from 121 women. The median $T_{pot}$ was 4.0 days. More rapid proliferation was seen in association with advanced clinical stage ($P=0.01$). Higher BrdUrd labelling was seen in association with pelvic tumour recurrence, this was the only biological/histological parameter with univariate and multivariate significance in relation to loco-regional recurrence ($P=0.006$ and $P=0.034$ respectively).

This study represents the first assessment of $T_{pot}$ in relation to radiotherapy treatment response of cervical carcinoma. The association of rapid proliferation and poor pelvic disease free survival indicates the need for further research into the potential of radiotherapy schedule alteration to reflect tumour proliferation. The predictive value may be enhanced by combination with other biological parameters.

### 1.3 Stable bioenergetic status during severe hypoxia in a mouse tumour

M Nordsmark[1]*, RJ Maxwell[2], MR Horsman[1] and J Overgaard[1]

*[1]Danish Cancer Society, Dept. of Experimental Clinical Oncology[2], Centre for Magnetic Resonance, Aarhus University Hospital, Denmark.*

**Background:** Hypoxic cells in solid tumours are known to be relatively radioresistant (1,2). Therefore several attempts to identify radiobiological hypoxia have been suggested, among these $pO_2$ electrode estimates of oxygenation status and $^{31}P$ MR Spectroscopy ($^{31}P$ MRS) (3–4). Recent studies have however questioned the susceptibility of energy measurements as indicator of hypoxia (5–7). We here report results of gas breathing induced changes on tumour $pO_2$ and energy status.
**Materials and methods:** A C3H mammary carcinoma grown in the feet of CDF1 mice was used. Non-anaesthetized mice were restrained and allowed to breath either 100% oxygen, carbogen (95% $O_2$+5%$CO_2$), carbon monoxide (CO) at 660 p.p.m. or atm. air for 64 min. Bioenergetic status was measured continuously by $^{31}P$ MRS (7 Tesla SISCO spectrometer) and evaluated as beta nucleosidetriphosphate/inorganic phosphate ($\beta$-NTP/Pi) ratio. Tumour oxygenation was measured polarographically (Eppendorf, Germany) after either 16 or 45 min and evaluated as *the percentage of* $pO_2 \leqslant 5$ *mmHg*.
**Results:** Carbogen and 100% oxygen breathing resulted in a continuous and significant increase in the $\beta$-NTP/Pi ratio. This was compatible with the reduction in hypoxia from 61% to 32 *percent of* $pO_2 \leqslant 5$ *mmHg* respectively. Breathing of CO 660 p.p.m. however, increased the *percentage of* $pO_2 \leqslant 5$ *mmHg* from 61 to 94%, whereas the $\beta$-NTP/Pi ratio was stable over 64 min.
**Conclusion:** Tumour cells *in vivo* were able to maintain the bioenergetic status during a period of at least 1 h of severe hypoxia – almost equivalent to the level of oxygenation achieved by clamping of the blood supply.

**References**

1. MOULDER. (1984). *Int. J. Radiat. Oncol. Phys.*, **10,** 695.
2. OVERGAARD. (1989). *Int. J. Radiat. Oncol. Phys.*, **56,** 801.
3. KALINOWSKI. (1990). *Int. J. Radiat. Oncol. Phys.*, **19,** 953.
4. CHAPMAN. (1991). *Radiother. Oncol.*, suppl. 20 **13,** 20.
5. GERWECK. (1993). *Radiother. Res.*, **135,** 69.
6. VAUPEL. (1994). *Br. J. Cancer.*, **69,** 46.
7. NORDSMARK. (1995). *Acta Oncol.*, **34(3),** 329.

Supported by a grant rom the Danish Cancer Society.

### 1.4 Comparison of effects of nicotinamide and pentoxifylline on RIF-1 tumour oxygenation

DJ Honess*, MS Andrews and NM Bleechen

*Univ. and MRC Dept. of Clinical Oncology, Hills Rd, Cambridge, CB2 2QH, UK.*

Pentoxifylline (PXF) and nicotinamide (NCT) are effective radiosensitisers of murine tumours which act by increasing tumour $pO_2$. NCT is known to reduce intermittent hypoxia and PXF appears to act similarly. We have studied their effects on the 600 $mm^3$ RIF-1 tumours with the aim of comparing the magnitude and time course of the effects of both agents. The $pO_2$ distribution was measured with the Eppendorf 6650 histograph system with a 0.7 mm net forward step for each reading, 50-60 readings per tumour and 10-12 tumours per group. The $pO_2$ distribution is highly skewed to the lower values and was described by the median and the percentage of values <2.5 mmHg, i.e. those thought to represent biologically relevant hypoxia. Control medium $pO_2$ was ~1 mmHg, with ~80% values of <2.5 mmHg. Maximum increases in $pO_2$ after PXF were at 15 min after 20 mg/kg PXF (mean±2SE of median: 15±2 mmHg; mean of % <2.5 mmHg: 19±3 and after NCT were at 1 h after 250 mg/kg NCT medium 24±3 mm Hg, 7±3% <2.5 mmHg). The half-times for decay of these changes ($t[pO_2]_{½}$) were calculated from $pO_2$ measurements at appropriate times after the peak effect. PXF $t[pO_2]_{½}$ was 4.3 (3.4-5.9) min for the median value which corresponds closely with the plasma half-life ($t_{½}$) of PXF, 4.6 (4.2-2.1) min but $t[pO]_{½}$ was significantly longer for the % <2.5 mmHg at 9.9 (7.3-15.5) min. NCT $t[pO_2]_{½}$ was 3.2 (2.4-5.0) h for the median value, slightly longer than the plasma $t_{½}$ of NCT i.e. 2.2 (2.0-2.4) h and again was significantly longer for the % <2.5 mmHg at 9.4 (6.3-19.2) h. The fact that $t[pO_2]_{½}$ for median values for both drugs was comparable with plasma $t_{½}$ was consistent with the requirement for the drug to be present for $pO_2$ modification. It follows that the longer the drug availability, the greater the likely magnitude of $pO_2$ change. The greater effect on $pO_2$ by NCT than PXF could therefore be attributable to its longer plasma $t_{½}$. This hypothesis was tested by prolonging the plasma availability of PXF by giving 20 mg/kg followed by 2×10 mg/kg at

10 min intervals (40 mg/kg in all), and measuring $pO_2$ 15 min after the final dose. The $pO_2$ increase (median $21 \pm 2$ mmHg, $8 \pm 3\%$ <2.5 mmHg) was larger than that for 40 mg/kg as a single dose (median $15 \pm 2$ mmHg, $16 \pm 4\%$ <2.5 mmHg) which is isoeffective with 20 mg/kg as a single dose (median $15 \pm 1$ mmHg, $17 \pm 4\%$ <2.5 mmHg). These results are consistent with the hypothesis. The half-times for decay of the effect of the prolonged PXF dosing were measured and $t[pO_2]_{1/2}$ was increased to 10.0 (7.3-15.5) min for median values and to 15.0 (12.1-20.0) min for % <2.5 mmHg. We conclude that extending the drug availability increases the magnitude of the $pO_2$ increase and prolongs the $t[pO_2]_{1/2}$ for the % <2.5 mmHg.

### 1.5 The effect of carbogen breathing on intra- and extracellular pH and [lactate] in rat hepatomas

M Stubbs*, SP Robinson, LR Rodrigues and JR Griffiths

*Cancer Research Campaign Biomedical MR Research Group, Division of Biochemistry, St George's Hospital Medical School, London SW17 ORE, UK.*

It has been known for many years that tumours produce large amounts of lactic acid and recently it has been shown that the intracellular pH ($pH_i$) of rodent tumours is higher than that of the tumour extracellular fluid ($pH_e$), in contrast to normal tissues (e.g. liver) in which $pH_i$ is lower than $pH_e$ (Stubbs *et al.*, 1994 *Cancer Res.*, **54**, 4011). We have used carbogen (95% $O_2$/5% $CO_2$) breathing to alter the tumour environment to study the relationship between $pH_i$, $pH_e$ and [lactate] *in vivo* in Morris Hepatoma 9618A grown subcutaneously in rats. Measurements of blood $pCO_2$, $pO_2$, pH and $HCO_3^-$, were made by conventional analyses whereas $pH_i$ and $pH_e$ (using 3-aminopropyl phosphonate as an extracellular marker) of the hepatomas were made by $^{31}P$ Magnetic Resonance Spectroscopy followed by lactate measurements on tissue extracts. When hepatomas in normal air-breathing animals were compared to hepatomas from carbogen breathing animals the following was observed:

| *Time of breathing carbogen* | *$pH_i$* (n=4) | *$pH_e$* (n=4) | *lactate µmol/gm* (n=2-3) |
|---|---|---|---|
| 0 min | $7.00 \pm 0.17$ | $6.79 \pm 0.04$ | $4.9 \pm 0.9$ |
| 30 min | $6.99 \pm 0.17$ | $6.44 \pm 0.20$ | 2.0 |

These findings are interpreted in terms of the regulation of intracellular pH in tumours and show that even in the presence of large amounts of $CO_2$, $pH_i$ is maintained neutral by the tumours with compensating changes in other ions or compartments.

### 1.6 Tumour $pO_2$ predicts radiation response in mice and men

M Nordsmark*[1], M Overgaard[2], MR Horsman[1], J Overgaard[1]

*[1]Danish Cancer Society, Dept. Experimental Clinical Oncology and [2]Dept. Oncology, Aarhus University Hospital, Denmark.*

There is substantial evidence that hypoxic tumour cells compromise the effect of irradiation (1,2). In this report oxygenation status (tumour $pO_2$) was correlated with radiation response in 108 C3H mouse mammary carcinomas and in 36 patients with advanced squamous cell carcinomas of head and neck. Tumour $pO_2$ was measured by polarographic oxygen electrodes (Eppendorf, Germany) and evaluated as *the median $pO_2$, the percentage of $pO_2$ values $\leqslant 2.5$ mmHg and 5 mmHg*. In mice group one ($n = 52$) tumour $pO_2$ was hypothesized to predict outcome of radiation response. The median value of the three oxygenation parameters was related to the probability of tumour control after 55 Gy single dose and tumour control showed to be significantly lower among the 26 most hypoxic tumours. In mice group two ($n = 56$) the hypothesis was tested and fulfilled, *the percentage of $pO_2$ values $\leqslant 2.5$ mmHg* being the dominating parameter among the three in question. This endpoint was subsequently chosen for evaluation of the clinical data. Patients were treated by conventional external radiotherapy 66-68 Gy in 33-34 fractions. 16 patients had locoregional tumour recurrence. Among these 16 patients the median of *the percentage of $pO_2$ values $\leqslant 2.5$ mmHg* was 30 (range 0-95%) as compared to the 20 patients without failure, who had a median of 6 (range 0-51%). When separating all 36 patients by *the median* of *the percentage of $pO_2$ values $\leqslant 2.5$ mmHg* and comparing the actuarial tumour control at 2 years using Kaplan–Meier estimates the most hypoxic part had significantly lower tumour control ($p < 0.014$, Logrank test). Also by Cox multivariate analysis *the percentage of $pO_2$ values $\leqslant 2.5$ mmHg* as continous variable was found to be significant ($p = 0.0065$). In conclusion these results suggest that tumour oxygenation is predictive of radiation response.

#### References

1. MOULDER JE *et al.* (1984). *Int. J. Radiat. Oncol. Phys.*, **10,** 695.
2. OVERGAARD J. (1989). *Int. J. Radiat. Oncol. Phys.*, **56,** 801.

Supported by a grant from the Danish Cancer Society.

### 1.7 Adjuvant intraperitoneal radioimmunotherapy of ovarian cancer: a case-control report of phase II survival

S Nicholson*[1], C Gooden[1], V Hird[2], P Mason[2] and AA Epenetos[1]

*[1]ICRF Oncology Unit, Hammersmith Hospital, W12 0HS, [2]Dept. of Gynaecology, Sammaritan Hospital, London.*

Overall survival for advanced ovarian cancer remains poor, in spite of complete remission being obtained following debulking surgery and platinum-based chemotherapy. 25 patients received single-dose intraperitoneal radioimmunotherapy with yttrium-90-labelled HMFG1 murine monocloncal antibody, all in the adjuvant setting, i.e. once disease-free following chemotherapy.

We have sought to provide a meaningful assessment of the long-term survival of these patients by conducting a case-control study, with controls being drawn from the database of the North Thames Ovary Group. Controls were matched to cases on the basis of FIGO stage, age, histological grade and histological subtype. Where perfect matches could not be found, controls were selected with the best available prognostic variables. Closely-matched controls were found for 20 cases.

Survival at 5 years is 80% for cases compared to 55% for controls ($p = 0.0335$). Median follow-up is 59 months for cases compared to 27 months for controls. Median survival has been reached for neither group.

This treatment is now in Phase III trial. We would welcome referral of all eligible patients.

### 1.8 The morphology of mesenteric resistance arteries is altered in the presence of liver tumour in the rat

AM Wood*, MA Spence, WJ Angerson and TG Cooke

*University Dept. Surgery, Glasgow Royal Infirmary, Glasgow G31 2ER.*

The presence of hepatic tumour in both patients with colorectal liver metastases and in rat models of liver tumour is associated with decreased portal venous blood flow and increased splanchnic vascular resistance (Leen E *et al. Br. J. Surg.*, 1993; **80,** 1249; Hemingway DM *et al. Br. J. Surg.* 1991; **78,** 326). These changes are due, at least in part, to the presence of a circulating vasoconstrictor (Carter R *et al. Br. J. Cancer*, 1994; **69**, 1025). To determine whether these haemodynamic changes are also associated with structural changes in splanchnic vessels, we have measured wall dimensions in mesenteric resistance arteries from rats with and without hepatic tumours. The small bowel was isolated and harvested 23-28 days following intra-hepatic inoculation of either HSN sarcoma cells or a control solution and perfused via the superior mesenteric artery at 90-100 mmHg pressure, using a 2.5% buffered glutaraldehyde solution. The samples, taken from 13 tumour-bearing and 13 control rats, were coded so that the measurements could be performed in a blind manner. Tissue blocks were embedded in wax and sectioned so that the vessels of interest were cut in transverse section. The vessels were stained with haematoxylin and eosin and viewed using an image analysis system which allowed lumen diameter as well as median and wall thickness to be measured. Average media-to-lumen and wall-to-lumen ratios were calculated for each animal and compared for each group using a Mann-Whitney U test. Results are expressed as median value (interquartile range).

| | *Tumour* | *Control* | *p* |
|---|---|---|---|
| Media-to-lumen ratio | 0.062 (0.052–0.086) | 0.048 (0.042–0.060) | 0.031 |
| Wall-to-lumen ratio | 0.125 (0.105–0.174) | 0.090 (0.075–0.135) | 0.031 |

Media-to-lumen and wall-to-lumen ratios were significantly increased in tumour-bearing animals compared with controls, demonstrating that the presence of liver tumour can alter the structure of extrahepatic blood vessels. These findings are consistent with the changes in splanchnic blood flow which occur *in vivo* in liver cancer. Ongoing studies will determine whether increased wall thickness is due to smooth muscle hyperplasia or hypertrophy or to vessel remodelling.

### 1.9 Tumour angiogenesis in NSCLC

KJ O'Byrne, MI Koukourakis, A Giatromanolaki, R Whitehouse, DC Talbot, KC Gatter and AL Harris

*ICRF Clinical Oncology Unit and Dept. of Cellular Science, Oxford Radcliffe Trust*

Tumor angiogenesis is an important factor for tumor growth and metastasis. Although some recent reports suggest that microvessel counts in non small cell lung cancer are related to a poor disease outcome, the results were not conclusive and were not compared with other molecular prognostic markers. In the present study we assessed the vascular grade in 107 (T1,2-N0,1) operable non small cell lung carcinomas using the JC70 monoclonal antibody to CD31. Three different vascular grades were defined with appraisal by eye and Chalkley counting - high (Chalkley score 7-12), medium (5-6) and low (2-4) vascular grade. There was a significant correlation between eye appraisal and Chalkley counting ($p<0.0001$). Vascular grade was not related to histology, grade, proliferation index (Ki67), EGFR or p53 expression. Tumors from younger patients had a higher degree of angiogenesis ($p=0.05$). Apart from the vascular grade, none of the other examined factors was statistically related to lymph-node metastasis ($p<0.0001$). A univariate analysis of survival showed that vascular grade was the most significant prognostic factor ($p=0.0004$) followed by N-stage ($p=0.001$). In a multivariate analysis, N-stage and vascular grade were not found to be independent prognostic factors since both were strongly related to each other. Excluding N-stage, the vascular grade was the only independent prognostic factor ($p=0.007$). Kaplan-Meier survival curves showed a statistically significant worse prognosis of patients with high vascular grade. No difference was observed between low and medium vascular grade. Our data suggest that angiogenesis in operable non small cell lung cancer is a major prognostic factor for survival and, among the tested parameters, is the only factor related to cancer cell migration to lymph-nodes. We are currently evaluating the relationship of angiogenesis to Bcl-2, c-erbB-2 and thymidine phosphorylase expression in NSCLC. The integration of vascular grading in clinical trials on adjuvant chemotherapy and/or radiotherapy may substantially contribute to defining groups of operable patients that would benefit from cytotoxic treatment.

### 1.10 A method for the generation of murine monoclonal antibodies with specificity for rat tumour vasculature

NJ Bradley, CT Baillie and MC Winslet

*University Department of Surgery, Royal Free Hospital Medical School, Pond Street, London NW3 2QG.*

The demonstration of proliferation antigens discriminating tumour from normal endothelium, implicates tumour endothelium as a potential target for antibody-mediated immunotherapy. The aim of this study was to generate tumour vascular-specific monoclonal antibodies (MoAbs) for testing in an *in vivo* tumour system.

A spontaneous transplantable renal carcinoma arising in the BALB/c mouse (RAG cell line) was grown subcutaneously in Chester Beatty Hooded nude rats. The resulting tumours were implanted subcutaneously or intraperitoneally in 7 BALB/c mice as 1-3 $mm^3$ fragments (Home Office approved), inducing an anticipated immune response to the rat tumour stromal component alone. Tumours were implanted after rapid freeze/thawing to produce tumour cell death. Two further tumour implants were performed, at monthly intervals.

Hybridomas were generated by the method of Kohler and Milstein. Supernatants were screened for the presence of antibodies by indirect immunoperoxidase staining on 6 mm frozen sections of RAG tumour and a normal tissue multiblock from the nude rat. Three hybridomas produced antibodies with tumour vascular, with 2 successfully cloned by serial limiting dilutions. The $IgG_1$ MoAb, 003, has stained the vasculature in 10 different individual rat tumours with minimal cross-reactivity in normal tissues, confirming the potential of this methodology to generate tumour vascular-specific MoAbs.

### 1.11 Increased expression of PAI-1 constructs in tumour-stimulated endothelial cells

SG Martin, PW Hewett, J Carmichael and JC Murray

*University of Nottingham Laboratory of Molecular Oncology, CRC Department of Clinical Oncology, City Hospital, Nottingham NG5 1PB, UK.*

An alternative to the more conventional approach of targeting toxic agents to tumour cells, is to take advantage of differences between normal and tumour-associated endothelium. We are investigating the use of tissue-specific promoters to target therapeutic genes to tumour-stimulated endothelial cells. Plasminogen activator inhibitor-1 (PAI-1) plays an important role in maintaining the normal homeostatic balance in blood and has been shown by *in situ* hybridisation to be up-regulated in tumour-associated endothelium. We initially used RT-PCR to investigate expression of the PAI-1 gene in human umbilical vein endothelial cells (HUVEC) and mammary fat microvessel endothelial cells (HUMMEC) under a variety of conditions. Expression was down-regulated in quiescent compared to proliferating endothelial cells. However, PAI-1 could be induced in quiescent HUVEC and HUMMEC cultures by exposure to medium conditioned by a number of tumour cell lines (TCM). We then attempted to transfect HUVEC with the pOCAT Hind plasmid, containing a 826 bp fragment of the PAI-1 promoter coupled to a CAT reporter gene (kindly supplied by Dr A Belayew, Katholieke Universiteit Leuven, Belgium). Of a variety of techniques used, only electroporation was successful, yielding reproducible results. A 72 h expression period was allowed following transfection and HUVEC were cultured in TCM or control medium for a further 5 h. TCM enhanced CAT production approximately 3-fold as measured by CAT-ELISA. We are currently examining the effects of TCM from a range of solid tumours on HUMMEC as well as HUVEC transfected with pOCAT Hind.

### 1.12 Bone mineral density in adults with lymphoma and the effects of treatment

E Brankin*[1], SJ Gallacher[2], R Bessent[2], M Soukop[1], IT Boyle[2]

*[1]Dept. of Medical Oncology, [2]University Dept. of Medicine, Glasgow Royal Infirmary, 10 Alexandra Parade, Glasgow, G31 2ER.*

Bone density was measured by DXA (Lunar DPX) at lumbar spine (L2-4) and neck of femur (NOF) in 19 males (mean age 41 years, range 18-59) peviously treated by radiotherapy (n = 5) or chemotherapy (n = 14) for lymphoma (mean 9.5 years from diagnosis, range 3-15) and compared with 29 age/sex matched controls. Bone density tended to be lower at L2-4 at (mean ± SEM) 1.180 ± 0.043 g/cm$^2$ versus 1.234 ± 0.016 g/cm$^2$ (p = NS) (median Z-score = 0.5, range −1.8 to 4.0) and was lower at NOF (0.982 ± 0.033 g/cm$^2$ vs 1.075 ± 0.025 g/cm$^2$, p = 0.03, median Z score = −0.03 range −1.9 to 2.8). A further 9 patients (8 females, mean age 52 years) were followed prospectively from lymphoma diagnosis. Over a mean follow-up interval of 19 months L2-4 fell from 1.112 ± 0.104 g/cm$^2$ to 1.047 ± 0.106 g/cm$^2$ [−4.1 ± 0.9%/year], p = 0.008 and NOF from 0.976 ± 0.080 g/cm$^2$ to 0.913 ± 0.79 g/cm$^2$ [−4.6 ± 1.0%/year], p = 0.0002. Percentage fall/year was greater in these patients at both L2-4 and NOF than in group of 52 control subjects (−0.6 ± 0.3%, p = 0.001 and −0.3 ± 0.3%, p = 0.004 respectively). These results suggest patients with lymphoma are at risk of accelerated bone loss around the time of diagnosis after treatment of lymphoma and of osteoporosis in later years.

### 2.1 Familial predisposition to both male and female germ cell tumours?

RA Huddart*[1,2], C Thompson[1], EJ Nicholls[2], A Horwich[2] and R Houlston[1]

*[1]Institute of Cancer Research, 15 Cotswolds Road, Belmont, Surrey SM2 5NG and [2]The Royal Marsden Hospital, Downs Road, Sutton, Surrey.*

A minority of testicular teratomas are recognised to be familial [Forman *et al.* Br. J. Cancer **65** p 255–262]. Some occur as part of the spectrum of cancers in the Li-Fraumeni syndrome, however, the genetic basis of the majority of familial cases is unknown. This has prompted the formation of a Linkage Consortium to identify genes causing testicular teratomas. As one of the participating centres we have been ascertaining familial cases from a registry of testicular teratoma patients at the Royal Marsden Hospitals. Amongst these cases we have identified 3 families which suggest that a common genetic basis exists between some male and female germ cell tumours.

The first family was identified through an index case who presented with a seminoma at age 51, his brother had had a testicular teratoma at age 28 and their cousin an endodermal sinus tumour of the ovary diagnosed at 32. In the second family the index case presented with an undifferentiated malignant teratoma at 28 years of age and his sister was diagnosed with bilateral mature teratomatous cysts at age 39. In the third family the index case presented with a retroperitoneal teratoma at 26 and his sister was diagnosed with an ovarian dysgerminoma at 45. None of these families had any features indicative of the Li-Fraumeni syndrome or any other cancer family syndrome suggesting the identification of a previously unrecognised association. The 3 families we report were identified from a database of 2000 teratoma patients, suggesting that in 0.2% of pedigrees a female member will develop a germ cell tumour. This may be an underestimate since pedigree information on all 2000 index cases has not been verified and many mature teratomatous cysts are asymptomatic and go undiagnosed.

Whether an association between male and female germ cell tumours is due to the inheritance of a single gene with effects on both ovary and testis or a consequence of the action of modifying genes will only be established when the gene or genes causing testicular teratomas are identified.

This work is supported by the Cancer Research Campaign and the Bob Champion Cancer Trust. RAH is supported by a CRC clinical research fellowship.

### 2.2 Socioeconomic deprivation and colorectal cancer incidence in N. Ireland

RH Wilson*[1], F Kee[2], C Patterson[2], S Currie[3], JM Sloan[4], RF Houston[1], BJ Rowlands[5] and RJ Moorehead[5]

*N. Ireland Centre for Clinical Oncology[1], Departments of Public Health Medicine[2], Pathology[4] and Surgery[5], The Queens' University of Belfast, Belfast and IT Department, Northern Health and Social Services Board[3].*

Colorectal cancer (CRC) shows a wide variation in incidence worldwide. Studies of migrant populations suggest that much of the cross-cultural variation in incidence may be attributable to environmental factors. Aetiological factors investigated included diet, sedentary lifestyle, body mass index and habitual alcohol or coffee consumption. The OPCS Longitudinal Study did not show a link between CRC incidence and social factors. N. Ireland has the highest degree of social polarisation of any region within the UK.

The aim of this study was to determine whether CRC incidence differed in areas of high and low socioeconomic deprivation in N. Ireland.

The Northern Ireland Colorectal Cancer Register provided details on all the 1178 newly-diagnosed patients with CRC in our province in 1990 and 1991. We related age, sex, site and Dukes' stage to levels of socioeconomic deprivation. Electoral wards were grouped into quintiles of the population after ranking by the Townsend Deprivation Score. The association between CRC incidence and Townsend Score was studied using Poisson regression analysis.

Age-standardised CRC incidence ranged from 22.5 to 29.9 per 100,000 for men from the least to the most deprived fifth of the population. The trend for females was similar but tended to plateau for the lower socioeconomic quintiles. After adjusting for age and sex, there was a significant association between CRC incidence and socioeconomic deprivation (p = 0.002). The rate ratio for the most to the least materially deprived quintile was 1.28 (95% CI 1.06–1.53). The effect was stronger for rectal (rate ratio 1.09) than colonic cancers (rate ratio 1.05). There was no statistically significant difference in the proportion of early and advanced Dukes' stage tumours between patients from the relatively affluent and the deprived areas.

We conclude that CRC incidence in N. Ireland is related to community deprivation. Further study is necessary to determine mechanisms for this association.

**Keywords:** Colorectal cancer, socioeconomic deprivation, cancer register

### 2.3 Dose-response study of superficial bladder cancer to intravesical administration of epirubicin

*[1]JRW Masters, [2]RJM Popert, [2]J Goodall, MJ Coptcoat and [3]MKB Parmar

*[1]University College London, Institute of Urology and Nephrology, 3rd Floor, 67 Riding House St, London W1P 7PN, [2]Department of Urology, King's College Hospital, Denmark Hill, London SE5 9RS and [3]MRC Cancer Trials Office, 1 Brooklands Avenue, Cambridge CB2 2BB.*

The aim of this study was to determine if a dose-response can be observed in the intravesical treatment of superficial bladder cancer with epirubicin. 100 patients with primary or recurrent pTa or pT1 disease were treated surgically, leaving a marker tumour in the bladder. Following histopathological confirmation of tumour stage, patients were randomized by the method of sealed envelopes to receive 1 mg/ml epirubicin (50 mg in 50 ml saline) or 2 mg/ml epirubicin (100 mg in 50 ml saline) between 10 and 21 days post-operatively. 3 months later the response of the marker tumour was assessed as complete (no tumour visible either cystoscopically or histopathologically in a biopsy taken at the site of the marker tumour) or as no response (histopathologically confirmed persistence of tumour). The volume of the bladder contents was measured after treatment, and taking into account dilution by urine the mean doses received were 0.7 mg/h/ml (1 mg/ml dose) and 1.4 mg/h/ml (2 mg/ml dose). 43/100 (43%) of the patients responded to the single instillation of epirubicin. Of the patients receiving 1 mg/ml epirubicin, 22/50 (44%) responded, compared to 21/50 (42%) of patients receiving 2 mg/ml epirubicin. 5 patients receiving the higher dose experienced bladder irritability to the extent that the instillate could not be retained for the full one hour.

This study does not provide any indication of a dose-response of superficial bladder cancer to epirubicin.

### 2.4 Effect of chemotherapy on the inflammatory response in patients with colorectal liver metastases

HJ Gallagher*[1], DC McMillan[1], A Rumley[2], G Lowe[2], TG Cooke[1] and CS McArdle[1]

*Departments of Surgery[1] and Medicine[2], The Royal Infirmary, Glasgow.*

Recent work suggests that the milieu in which the metastatic cell finds itself is crucial to its survival (Frost P, 1992, Lancet, 339: 1458–1461). It has been reported that many patients with advanced disease have an ongoing inflammatory response and there is increasing evidence that this promotes tumour progression. Hepatic arterial 5FU chemotherapy may delay or reverse tumour progression in patients with colorectal liver metastases. However, it may also result in tissue damage and thus enhance the inflammatory response. The overall effect on the inflammatory response has not been demonstrated. This was examined in a cohort of patients with colorectal liver metastases. Patients, of equivalent staging, were grouped according to whether they had yet received hepatic arterial 5FU chemotherapy ($n=13$) or not ($n=8$). Venous samples were centrifuged at 5°C and stored at −70°C. Plasma viscosity, fibrinogen, fibrin degradation products (FDP's) and interleukin-6 were measured. Results are given in median (range). Statistical comparisons were carried out using the Mann-Whitney U-test.

| | *No chemotherapy* | *Chemotherapy* | *p-value* |
|---|---|---|---|
| Plasma viscosity (mPa.s) | 1.50 (1.21–1.61) | 1.37 (1.21–1.57) | 0.17 |
| Fibrinogen (g/l) | 4.4 (3.1–6.2) | 4.4 (3.2–5.6) | 0.310 |
| FDP (μg/l) | 602 (118–1032) | 144 (75–592) | 0.003 |
| Interleukin-6 (pg/ml) | 15.7 (1.7–24.7) | 3.8 (0.81–24.5) | 0.02 |

There was a significant reduction in interleukin-6 and FDP concentrations in the patient group who had received chemotherapy. These results suggest that the contribution to the inflammatory response from this chemotherapy is small compared with that of the tumour. It may be that the changes in such inflammatory markers may provide a method of predicting response to chemotherapy.

### 2.5 Peripheral blood progenitor cell (PBPC) rescue following high dose chemotherapy in breast cancer: satisfactory early haematopoietic reconstitution

M Eatock*[1], G Cook[2], IM Franklin[2], P Tansey[2], M Soukop[1] and D Dunlop[1]

*Departments of Medical Oncology[1] and Haematological Oncology[2], Solid Tumour Transplant Programme, Glasgow Royal Infirmary University NHS Trust, Glasgow, G4 0SF.*

The existence of a dose response relationship in the treatment of breast cancer is supported by *in vitro* data and by clinical data suggesting an increased response rate for patients treated with high dose chemotherapy. A survival benefit has yet to be convincingly demonstrated for patients with advanced disease, however this technique may be useful in the adjunctive treatment of patients at high risk of relapse following intital surgery.

14 patients with breast cancer stage II-IV (4 with metastatic disease and 10 receiving adjuvant therapy for high risk disease), median age 43 years (range 28–55) underwent a high dose chemotherapy procedure with PBPC rescue. Of the 10 patients receiving adjuvant therapy, the median number of involved axillary lymph nodes was 6 (range 4–11 and all had T2 or T3 tumours).

Induction therapy consisted of cyclophosphamide (Cy)/Epirubicin (1 patient); 5-fluorouracil/epirubicin/Cy (FEC) (11 patients); doxorubicin (1 patient); doxorubicin followed by intermediate dose cyclophosphamide (1 patient). A mean of $3.28\times10^6$/Kg (range $1.46-7.75\times10^6$/Kg) CD34+ cells and $121.73\times10^4$/Kg (range $9.42-305.4\times10^4$/Kg) CFU-GM growth were harvested.

7 patients received conditioning therapy with high dose carboplatin, etoposide and melphalan (CEM), the remainder received Cy and thiotepa. PBPC rescue was given with a mean CFU-GM dose of $139\times10^4$/Kg (range $52.9-268\times10^4$/Kg). Median time to neutrophil engraftment ($>0.5\times10^9$/L) was 11 days (9–15 days) and median time to platelet engraftment ($>20\times10^9$/L) was 12 days (6–19 days). Median in-patient hospital stay following PBPC administration was 19 days (range 15–27 days). Pyrexia was observed in all patients with a median duration of 6.5 days (4–13 days) and WHO grade 3 or 4 gastrointestinal toxicity was observed in 10 patients. There were no procedure related deaths in this group of patients.

Of those patients treated with metastatic disease 3 have relapsed at 12, 6 and 3 months following PBPC rescue. No patient receiving adjuvant high dose therapy has relapsed to date with a median follow up of 14.3 months (5–22 months).

In our experience this is a safe procedure with no treatment related mortality. Despite encouraging initial reports of cure rates of up to 25% it remains to be proven that this type of therapy prolongs survival in patients with advanced breast cancer. The role of this treatment in the adjuvant situation is currently the subject of randomised trials in Britain and Europe.

### 2.6 Primary high dose dacarbazine in high grade glioma

AJ Salisbury*[1], M Saunders[2], AL Harris[3], SA Kyrtopoulos[4], AC Jones[1]

*[1]Radiotherapy Dept., Churchill Hospital, Oxford, OX3 7LJ; [2]MRC Radiobiology Unit, Chilton, Didcot, OX11 0RD; [3]ICRF Clinical Oncology Unit, Churchill Hospital; [4]Nat. Hellenic Res. Found., Inst. of Biol. Res. and Biotech., Athens 11635, Greece.*

The prognosis for glioblastoma mutliforme is extremely poor. Even with surgery and radiotherapy (RT), the one-year survival is 10–20%. Long term survivors are rare.

Dacarbazine (DTIC) has been used in the past with a response rate comparable to the nitrosoureas but at the cost of greater toxicity, principally vomiting. With the advent of $5HT_3$ antagonists, the emetic potential of DTIC was greately curbed. The *in vivo* activity of dacarbazine is dependent on its activation to MTIC, which methylates DNA producing $O^6$ and $N^7$ methylguanine. $O^6$-MeG has the major cytotoxic effect and is repaired by the suicide enzyme $O^6$-alkyl guanine DNA alkyl transferase ($O^6$-AGT). Dacarbazine and temozolamide are converted to the same active metabolite (MTIC). In view of the promising initial results of temozolamide in gliomas, it seemed appropriate to investigate the use of dacarbazine prior to RT in the treatment of high grade gliomas.

21 patients with glioblastoma multiforme and performance status 0-2 received dacarbazine 1 g per $m^2$ as a one hour infusion every three weeks. Response was assessed every 2 cycles both clinically and radiologically (CT or MRI). Patients received a maximum of 6 cycles of dacarbazine prior to RT. The mean age was 55 years (range 40 to 67), with a preponderance of male patients (17:4). All had histological diagnoses made, with specimens obtained at surgery (described as either a biopsy (10 patients), debulking (10 patients), or a macroscopic clearance (1 patient). All patients had their performance and neurological status recorded as well as standardised assessments of quality of life, anxiety and depression. During the first 2 cycles of treatment, enzyme ($O^6$-AGT) and adduct ($O^6$-MeG) levels in peripheral blood samples were measured.

For 17 of the 21 patients, there is follow up data for at least 6 months. 2 patients had a partial response (WHO criteria), and 1 patient had a 'significant' radiological response - but, as is common in glioma, formal 2-dimensional measurements were not possible because of the diffuse nature of the tumour. The mean progression free survival (PFS) of these patients was 527 days (613, 600, 369 days). Patients who maintained stable disease for three months during treatment (3 patients) have a considerably higher mean PFS to date than those who progressed on chemotherapy (195, 230 and 343 days, mean 256 days vs 46 days). 35% of patients in the study achieved either a partial response or stable disease. It is well established that the best results are seen in young patients of good performance status, in whom it is possible to remove most or all of the tumour. Our results are compatible with this.

A previous study using dacarbazine in melanoma demonstrated peak adduct levels 1–4 h after treatment, with prolonged reduction in the level of AGT. The enzyme and adduct levels are being analysed by Dr Kyrtopoulos and will be presented with the response and toxicity data.

No physiological morbidity was detected due specifically to the chemotherapy (using HAD and Rotterdam questionnaires). This regimen can be given as an outpatient, with appropriate anti-emetics.

In future studies it may be possible to schedule methylating agents in such a way to reduce the level of AGT to allow maximum $O^6$-MeG formation. This may be reflected by increased tumour response.

### 2.7 High dose therapy (HDT) with melphalan (M) and total body irradiation (TBI) or busulphan (B) for Ewing's sarcoma (ES)

JS Whelan* and RL Souhami

*The London Bone Tumour Service, Meyerstein Institute of Oncology, UCL Hospitals, London, UK.*

The prognosis of metastatic or recurrent ES is poor with conventional dose chemotherapy. Studies of HDT are difficult because of the rarity of ES. Thus, appropriate patients and therapy are as yet undefined. Ten patients have been treated with HDT and either bone marrow (3 patients) or peripheral stem cell rescue (PSCR, 6) or both (1). Median age was 16 years (range 9–30) and primary sites were femur, 4; pelvis, 3; humerus, 1; scapula, 1; extraosseous, thigh, 1. Lung metastases were present in 7 patients at diagnosis. Six of these patients had HDT as consolidation of response to first line induction treatment (3 pts) or second line therapy (3 pts). The 7th was treated when lung metastases recurred (1st remission, 35 months). Three further patients were treated at the time of a first recurrence locally (1; 1st remission, 13 mos) or in the lung (2; 1st remissions, 4 and 12 mos). Disease status at time of HDT was: 1st remission, 5; progressive disease, 1; 2nd remission, 4. HDT was Melphalan 110 mg/$m^2$ and TBI (3 patients) or Busulphan, $<16$ years, 600 mg/$m^2$; $>16$, 16 mg/kg (7). Cyclophosphamide, 1.5 mg/$m^2$ and Filgrastim 10 mg/kg was used for PSC mobilisation. Median number of days to neutrophils $>0.5\times10^9$/l was 13 (range 11–26) and to platelets $>20$, 19 days (10 →50). Impaired platelet recovery was frequent after M + B with PSCR. Other toxicities of M + B included seizures (2 pts), haemorrhagic cystitis (1), veno-occlusive disease (1) and pulmonary fibrosis (1). There were no treatment related deaths. Two patients have died of ES, 9 and 21 mos after HDT. Eight patients are alive without evidence of progression between 1 and 41 mos (median, 12) after HDT. Future studies will incorporate alternative agents. Multicentre studies are needed to aid selection of appropriate patients and thus determine any benefit of HDT in ES.

### 2.8 Use of high dose chemotherapy in adult Ewing's sarcoma and PNET

MW Verrill* and IR Judson

*Sarcoma Unit, Royal Marsden Hospital, Fulham Road, London SW3 6JJ.*

Adult patients (pts) with Ewing's Sarcoma (ES) have a poor prognosis which is thought to be due to almost invariable finding of bulky disease at presentation. Studies in paediatric patients suggest that melphalan containing high dose (HD) chemotherapy (CT) at first remission may improve survival in this group[1–3] and there are also reports showing high response rates in pts with a poor response to primary CT[4]. We have now treated 7 adult patients with bulky or metastatic ES with HD CT. Characteristics: median age 28 (17–42); all male; pelvis 3, thigh 1, chest wall 1, thumb 1, unknown, 1; metastatic 4, localised 3.

| Pt | 1° CT | Primary response | HD CT | Overall response | Time – progression | Time – death | Follow up |
|---|---|---|---|---|---|---|---|
| 1 | IVAD | PR | M | PR | 11 months | 18 m | |
| 2 | IVAD | NE | M | NE | 4 m | 6 m | |
| 3 | IVAD | PR | M | PR | 19 m | | 35 m |
| 4 | IVAD | NC | CM | PR | 7 m | | 21 m |
| 5 | IECG | PR | CEM | CR | | | 4 m |
| 6 | IECG | PR | CEM | CR | | | 2 m |
| 7 | IECG | PR | CEM | PR | | | 1 m |

IVAD = Ifosfamide/Vincristine/Adriamycin, IECG = Ifosamide/Etoposide/Cyclophosphamide/G-CSF
HD combinations: M = Melphalan, C = Carboplatin, E = Etoposide
Responses: Complete CR; Partial PR, No change NC, Not Evaluable NE.

We conclude that HD CT may produce disease stabilisation in pts with bulky tumours who achieve only PR after primary CT, this may lead to a survival benefit. Based on the paediatric data we hope that those pts who have achieved CR will show a similar trend. There is a need for multicentre national or international studies of HDCT in adults with Ewing's Sarcoma.

### References

1. MISER JS AND SANDERS JE. (1994). *Bone Marrow Transplant*, **14**, S54.
2. BURDACH ST, JURGENS H, PAPE H *et al.* (1994). *Bone Marrow Transplant*, **14**, S53.
3. BURDACH S, JURGENS H, PETERS C *et al.* (1993). *J. Clin. Oncol.*, **11**, 1482–1488.
4. MICHON J, HARTMANN O, DEMEOCQ F *et al.* (1994). *Proc. Annu. Meet. Am. Soc. Clin. Oncol.*, **13**, A1415.

### 2.9 A comparative study of subjective assessment, image analysis (IA) and ELISA for the quantification of cellular CEA levels

RCT Austin, NJ Bradley and MC Winslet

*University Department of Surgery, Royal Free Hospital Medical School, Pond Street, London, NW3 2QG.*

The use of immunocytochemistry for detection of cellular antigen is limited by the subjective quantification of immunostaining. Objective methods of assessment include computer aided image analysis and enzyme linked immunosorbent assay on whole cells (CELISA).

The aim of this study was to establish a CELISA for CEA and compare the results of subjective and objective quantification. Cytospin preparations ($10^6$ cells/ml) of two colorectal cancer cell lines (HT29, LoVo) with variable CEA expression, grown in the presence of augmenting agents (Theophylline/1e-2m, Butyric Acid/0.5e-3m and 5-Azacytidine/1e-6m) were processed by standard immunocytochemical techniques using the primary and secondary antibodies A5B7 and RAMP for subjective/image analysis. Identical cell preparations fixed to 96 well plates (Nunc) with 1% glutaraldehyde were blocked with 5% NHS and stained with A5B7 with optical density recorded at 450 nm on an ELISA plate reader. A subjective assessment of total cytospin ($10^5$ cells) were scored blind on a scale (0–12). Computerised video image analysis (Chromatic Colour Image Analysis System) recorded signal density per cell in arbitrary standardised unit with background standardisation.

Comparison of subject assessment and IA produced a coefficient of determination by regression analysis ($R^2$) of 0.415, whilst subjective analysis and CELISA produced a regression of 0.999. IA was strongly influenced by heterogeneous background illumination.

Subjective assessment of immunocytochemical CEA estimation is superior to objective image analysis and correlates closely with the results produced by independent standard CELISA.

### 2.10 Cell adhesion molecules in patients with TCC of the bladder: soluble serum E-cadherin correlates with predictors of recurrence

TRL Griffiths*[1,3], I Brotherick[3], RI Bishop[4], CHW Horne[4], BK Shenton[3], DE Neal[1,3] and JK Mellon[2,3]

*Depts. of Urology, [2]City Hospitals, Sunderland and [1]Freeman Hospital, Newcastle upon Tyne and Depts. of [3]Surgery and [4]Pathology, University of Newcastle, Newcastle upon Tyne, NE2 4HH.*

On newly-diagnosed superficial bladder tumours, approximately 30% are multifocal at presentation, 60–70% will recur and 10–20% will undergo stage progression. Multiplicity at presentation and a positive three-month check cystoscopy are factors associated with tumour recurrence. A proportion of cases with tumour recurrence may be due to dispersion of cells by mechanisms currently not well understood. It has been suggested that decreased intercellular adhesiveness favours detachment of tumour cells. Indeed, downregulation of E-cadherin expression on bladder tumours is associated with a reduced time to tumour recurrence. Soluble vascular cell adhesion molecules have been linked with angiogenesis in the rat model.

Sera from 40 patients with newly-diagnosed bladder cancer (28 superficial tumours [pTa and pT1] and 12 muscle-invasive tumours) and 13 control patients were assessed by ELISA to determine the concentrations of soluble cell adhesion molecules, sE-cadherin, sE-selectin, sVCAM-1 (vascular cell adhesion molecule-1) and sICAM-1 (intercellular adhesion molecule-1). Corresponding frozen sections of primary tumour were stained with a monoclonal antibody to E-cadherin, HECD-1, using standard immunohistochemistry.

Patients with bladder cancer had significantly higher concentrations of sE-cadherin compared with the control group ($P = 0.017$). No difference was found between the two groups with regard to sE-selection ($P = 0.403$), sVCAM-1 ($P = 0.942$) or sICAM-1 ($P = 0.092$). Levels of sE-cadherin were related to poor histological grade ($P = 0.011$), number of superficial tumours at presentation ($P = 0.008$) and a positive three-month check cystoscopy in superficial disease ($P = 0.036$). The lowest levels of sE-cadherin were found in patients who presented with solitary low grade tumours who had a negative three-month check cystoscopy. For those patients with muscle-invasive disease, elevated sE-cadherin was not related to the presence of metastatic lesions at presentation. Abnormal E-cadherin expression on the tumour was associated with stage ($P = 0.009$) and grade ($P = 0.03$). No correlation was found between high levels of sE-cadherin in sera and an abnormal E-cadherin staining pattern ($P = 0.077$).

Elevated levels of sE-cadherin can be detected in sera of patients with bladder cancer. Its association with multifocality at presentation and tumour recurrence suggest that it is a serum marker with potential predictive value in determining future tumour behaviour.

### 2.11 Tamoxifen inhibition of human melanoma cell invasion *in vitro*

LO Dewhurst*, M Wagner, IG Rennie and S MacNeil

*Depts. of Medicine, Clinical Sciences Centre, Northern General Hospital, Sheffield, S5 7AU.*

The need for tumour cells to interact with stromal extracellular matrix (ECM) proteins to migrate and invade, is an area for potential pharmacological intervention. In recent years, we have found evidence that both intracellular calcium and calmodulin appear to play important roles in the attachment of several normal and neoplastic cells to ECM proteins (e.g. as summarised in MacNeil *et al.*, 1994, Pigment Cell Research 7, p 222).

In this study, we have begun to examine the concept that inhibition of intracellular calmodulin activity will reduce tumour cell invasion though ECM proteins. We have initially used Tamoxifen, a drug with both anti-oestrogen and anti-calmodulin properties ($IC_{50}$ around 2 μM - MacNeil *et al.* (1993) Melanoma Research 3 p67).

Human cutaneous melanoma cells A375-SM, normal human uveal melanocytes and dermal fibroblasts at 1.2-$1.8 \times 10^4$ cells/well were added in serum-free media to the upper chamber of a Costar Transwell insert containing a 6.5 mm diameter polycarbonate filter with 8 μm diameter pores the underside of this filter, being coated with 0.5 μg of human fibronectin. The Transwells were placed in 24 well plates containing serum-free media and were cultured for 20 h with or without Tamoxifen. Trypsin/EDTA was used to remove cells from the underside of the filter and bottom of the 24 well, and from the upper chamber and upper surface of the filter. The invaded and non-invaded cells were then counted and % invasion calculated.

Under these conditions, fibroblasts were found to be non-invasive (in 3 out of 3 experiments), melanocytes found to be $4.7 \pm 2.66\%$ ($n=3$) invasive and A375-SM were $18.5 \pm 4.2\%$ ($n=8$). Tamoxifen significantly reduced both melanocyte and melanoma cell invasion with 50% reduction of invasion occuring at $0.4 \pm 0.2$ μM ($n=5$). The concentrations of Tamoxifen were not associated with any direct cytotoxic effect of the drug on the cells.

We conclude that Tamoxifen can reduce the ability of both normal (melanocyte) and neoplastic (melanoma) cells to interact with and invade through fibronectin. Such an action of Tamoxifen may be very relevant to its recently reported synergistic actions with other chemotheraputic agents in melanoma. It now needs to be determined whether Tamoxifen is acting as an anti-oestrogen or calmodulin antagonist.

### 2.12 Cyclin D1 expression and response to tamoxifen treatment for metastatic breast cancer

Diana M Barnes, Cheryl E Gillett, P Smith and RD Rubens

*ICRF Clinical Oncology Unit, Guy's Hospital, London.*

Cyclin D1 is a cell cycle associated protein active in the $G_1$ phase of the cell cycle. Amplification of the gene is found in approximately 20% of mammary carcinomas and immunohistochemistry has revealed that over-expression of cyclin D1 protein occurs more frequently. It is present in 40–50% of breast cancers suggesting oncogenic activity which could be associated with poor clinical outcome. Surprisingly, we found the reverse to be true; in primary breast cancer the highest levels of cyclin D1 expression occur in well differentiated ER positive tumours, usually associated with a good prognosis. We have also investigated the relationship between cyclin D1 protein expression and response to first line tamoxifen treatment for metastatic disease in 149 women. Response to treatment was assessed in a standard manner, according to UICC criteria and was available on all patients. Women whose response was unassessable were excluded from the study. 95 (64%) of cases overexpressed cyclin D1, 78 (82%) were ER positive. Response (complete/partial) was seen in 55 (71%) of these double positive tumours. Conversely tumours which were negative for both proteins had only an 8% chance of responding. Tumours which were positive for only one of the proteins had an intermediate response rate ($\chi^2 = 31.97$, $p < 0.0001$), suggesting that immunohistochemical staining for cyclin D1 could be a useful adjunct to the measurement in ER in identifying women who are likely to respond to endocrine treatment. Furthermore, these results pose interesting questions concerning the role of cyclin D1 in the biology of breast cancer.